# Artificial shaking signals in honey bee colonies elicit natural responses

**DOI:** 10.1038/s41598-020-60421-8

**Published:** 2020-02-28

**Authors:** Phoebe A. Koenig, Michael L. Smith, Logan H. Horowitz, Daniel M. Palmer, Kirstin H. Petersen

**Affiliations:** 1 Cornell University, Department of Electrical and Computer Engineering, Ithaca, 14850 NY United States; 2 Max Planck Institute of Animal Behavior, Department of Collective Behavior, Konstanz, 78464 Germany; 3 University of Konstanz, Department of Biology, Konstanz, 78464 Germany; 4 Centre for the Advanced Study of Animal Behaviour, University of Konstanz, Konstanz, 78464 Germany

**Keywords:** Social evolution, Behavioural ecology

## Abstract

Honey bee signals are primarily studied through natural observation combined with manipulations of the colony or environment, not direct manipulation of the signal stimulus or receivers. Consequently, we know little about which signal aspects are necessary to reproduce behavioral responses. Here, we focus on the shaking signal, wherein a worker grabs onto another bee and vibrates. All castes receive shaking signals, but individual responses depend on context, and the signal may be multi-modal (mechanical, odor, sound, etc.). We designed a tool to mimic the shaking signal. We tested whether a purely mechanical stimulus elicited the same behavioral response as a natural shaking signal, teasing apart the effects of signal and receiver characteristics. We found that both workers and drones increased their movement after being artificially shaken, and that shaken drones were more likely to engage in feeding and grooming than a sham control. These behavioral changes support the idea that the shaking signal serves to generally increase worker activity, but also serves to activate male reproductives (drones). With this tool, we show that vibration itself is responsible for eliciting much of the shaking signal’s behavioral response, in one of the few examples of direct playback in social insects.

## Introduction

Social insect colonies face the remarkable challenge of coordinating many individuals through decentralized systems to accomplish common goals. Colonies typically have castes or worker groups that perform different tasks, are located in different areas of the nest, and may respond to different stimuli, granting them experience-specific information about the broader colony context^1,2^. Social insects have evolved many signals that communicate specific information between individuals performing the same task^3^. One example is the honey bee waggle dance, which is performed by a forager bee to communicate the location of a good food source to another forager^4^. The honey bee shaking signal, however, is unique because it is used to relay *general* information to *all* workers, regardless of the task they are performing^5^. The shaking signal is considered a modulatory communication signal because it does not lead the recipient to change its behavior in a specific way. Instead, it leads to a general increase in activity by the receiver and an increased probability that they will behave in certain ways, depending on their identity^6^.

All workers can perform the shaking signal, even workers as young as 2 days old, but the majority of shaking signals are administered by older workers^7,8^. To perform a shaking signal, a worker bee grabs onto a recipient and vibrates at 16.3 ± 5.8 Hz for 1.2 ± 0.3 s^9^. While only workers transmit the shaking signal, all individuals in the colony can be recipients of the signal: workers, drones, and queens^6^. The response, however, varies depending on the recipient^10^. When workers are shaken, they increase their movement, change their trajectories within the nest, and those of foraging-age move to the dance floor (the area of the nest where successful foragers perform waggle dances to advertise foraging sites)^6,11^. When drones are shaken, they increase their movement, are fed and groomed more, and spend more time being fed and groomed than drones that are not shaken^12^. When a virgin queen is shaken, she is more successful at eliminating her rivals, and more likely to become the new queen of the colony than virgin queens that are not shaken^13^. When mated queens are shaken, they prepare to fly away with a swarm of workers and follow them to a new home^14^.

Peaks in shaking signal activity correlate with the time foragers began and ceased foraging on the previous 3-4 days, encapsulating the recent pattern of the forager work day, but peaks also occur when the colony finds forage after a famine^6,15^. The shaking signal is used so frequently and broadly that it is unlikely to be directed towards specific workers^16^. Schneider and Lewis (2004) proposed that the general meaning of the signal is "increase your activity”, where individual responses are produced by a combination of the recipient’s age, experience, physiological condition, genetically-influenced response thresholds, and other stimuli that may be present when the signal is received^5^.

Research on signal structure and function has relied heavily on experiments in which researchers present videos^17^, play song recordings^18^, induce vibrations^19^, or even present robotic animal mimics^20,21^ to assay behavioral responses. These types of experiments, which involve signal playback or mimicking, allow control of the stimulus and the receiver to tease apart the effects of different behaviors used in natural social interactions. Successful manipulation of an individual by playback or signal mimicry demonstrates a core understanding of the signal. Signal playback and mimicry enable one to reliably induce signals and observe responses, providing control over signal qualities and contexts which would otherwise be impossible.

In this study, we developed a stimulus tool to mimic the vibration that occurs during the shaking signal and tested it in observation hives against a sham control. The shaking signal is a vibro-tactile signal, but there are additional cues associated with it. For example, shaking is frequently performed by foragers after a successful trip, when they are still carrying nectar and pollen^6^. Our tool applies a vibration with the same duration and frequency as the shaking signal, testing whether the vibration alone elicits increased activity in worker and drone recipients. A research tool allowing the shaking of specific, individual bees provides the opportunity to study how response to the signal varies depending on characteristics of the receiver (e.g. caste, sex, age), the environment (e.g., time of day, season), and the colony (e.g. ontogeny, resource levels).

## Results

### Workers

**Figure 1:**
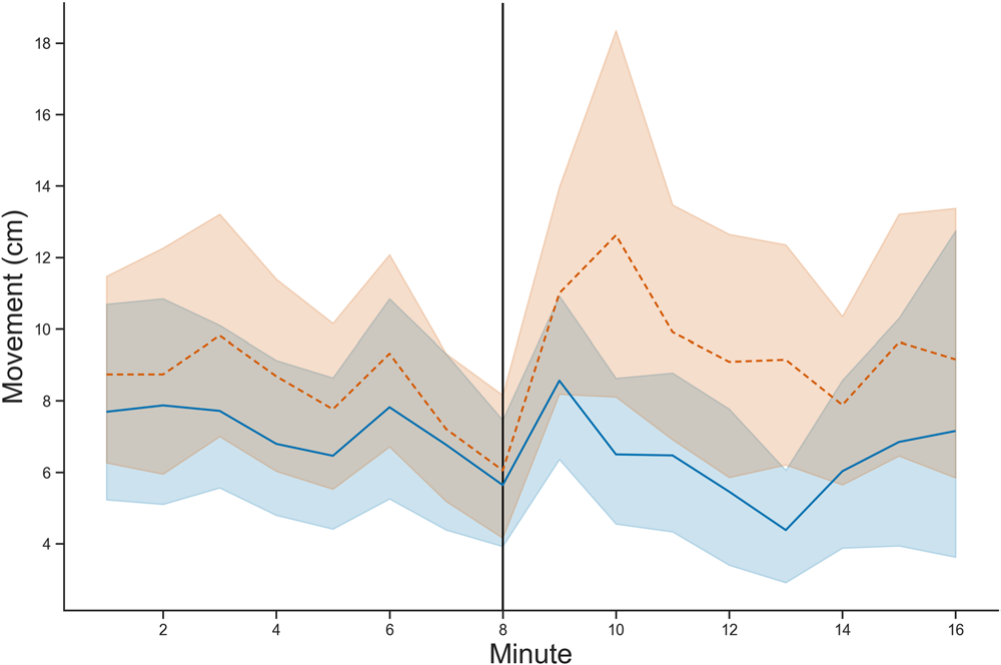
Movement of the shaken workers (orange dotted line, *n* = 38) and sham control workers (blue solid line, *n* = 39) during the 16 min observation. Intervention shown at minute 8 (vertical black line).

Shaken workers (*n* = 38) walked more than sham control workers (*n* = 39) after intervention with the magnetic shaker (lme, *p* = 0.036, Fig. 1). Shaken workers walked 9.34 ± 1.37 cm min^−1^ in the 8 minute observation period after intervention while sham control workers walked 6.39 ± 1.02 cm min^−1^. Values are reported as mean  ±  se unless stated otherwise. The difference in movement between shaken and sham control workers was greatest in the first 2 minutes following intervention, however, time since intervention was marginally non significantly (*p* = 0.057). In the first 2 minutes after intervention with the magnetic shaker, shaken workers walked 11.82 ± 1.83 cm min^−1^ while sham control workers walked 7.41 ± 1.04 cm min^−1^. In the last 2 minutes of the 8-minute observation period, shaken workers walked 9.39 ± 1.77 cm min^−1^ while sham control workers walked 6.70 ± 1.94 cm min^−1^. The difference in movement between the shaken and sham control workers persisted for the entirety of the observation period. The movement of each worker prior to intervention was a significant predictor of the bee’s movement after intervention (*p* < 0.001).

**Figure 2:**
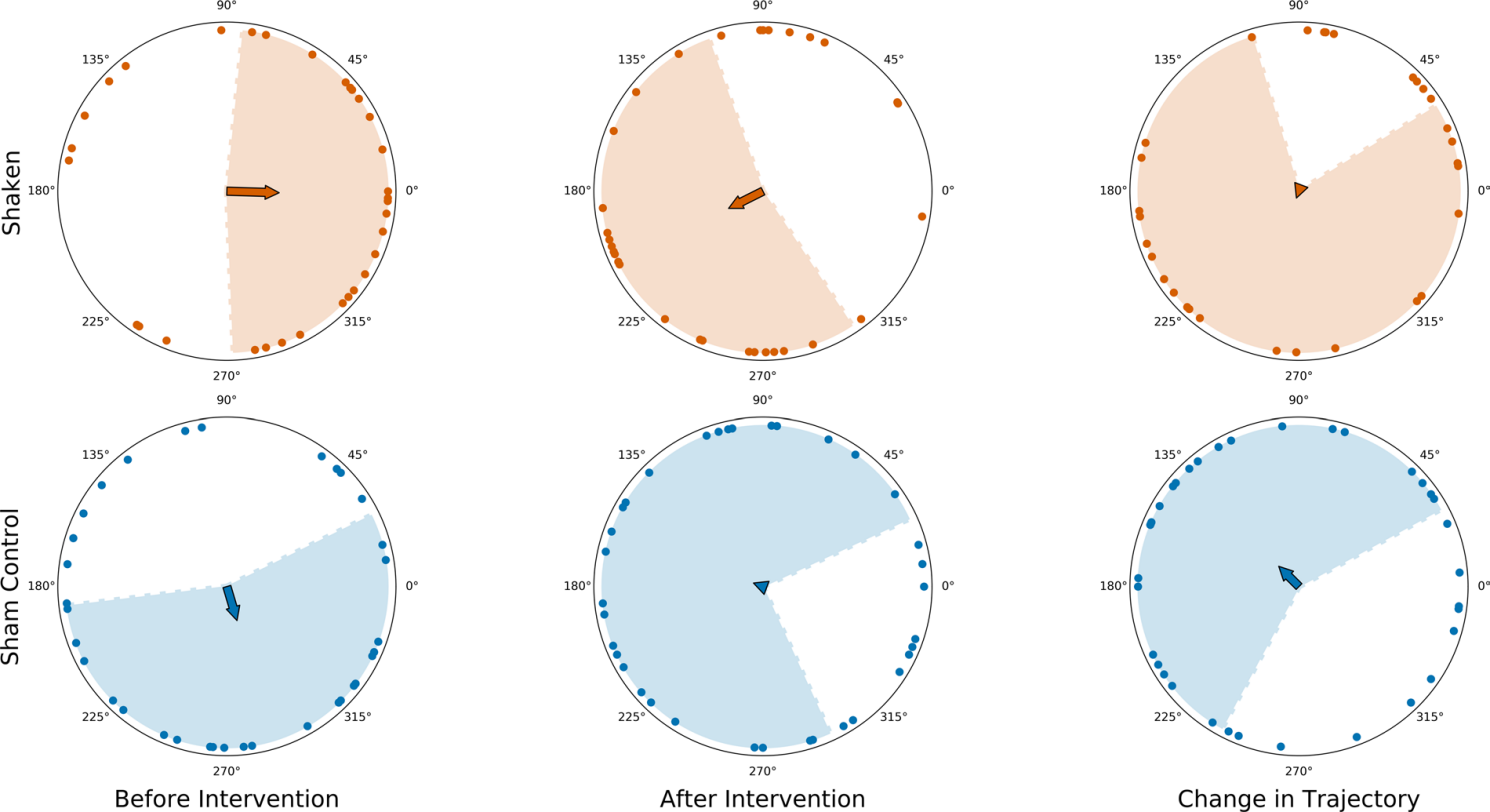
Trajectories of shaken workers (orange plots, top, *n* = 32) and sham control workers (blue plots, bottom, *n* = 36) before and after intervention, and the angle by which shaken and sham control workers changed their trajectory (i.e., the angle between their initial trajectory (min 1 − 8) and their trajectory after intervention (min 9 − 16)). Arrows show the magnitude and direction of the mean trajectory, shading denotes  ±  sd. Observation hive entrance is at 0 degrees.

We did not find any significant difference between the movement bearing of workers before intervention and their movement bearing after intervention in the shaken (*n* = 32) or sham control (*n* = 36) groups (Moore’s test, Shaken: *p* = 0.94, Sham control: *p* = 0.27, Fig. 2). We also looked at the angle by which each worker changed its bearing following intervention, and found no statistically significant difference in this angle between shaken and sham control workers (Watson two-test, *p* = 0.19).

### Drones

**Figure 3:**
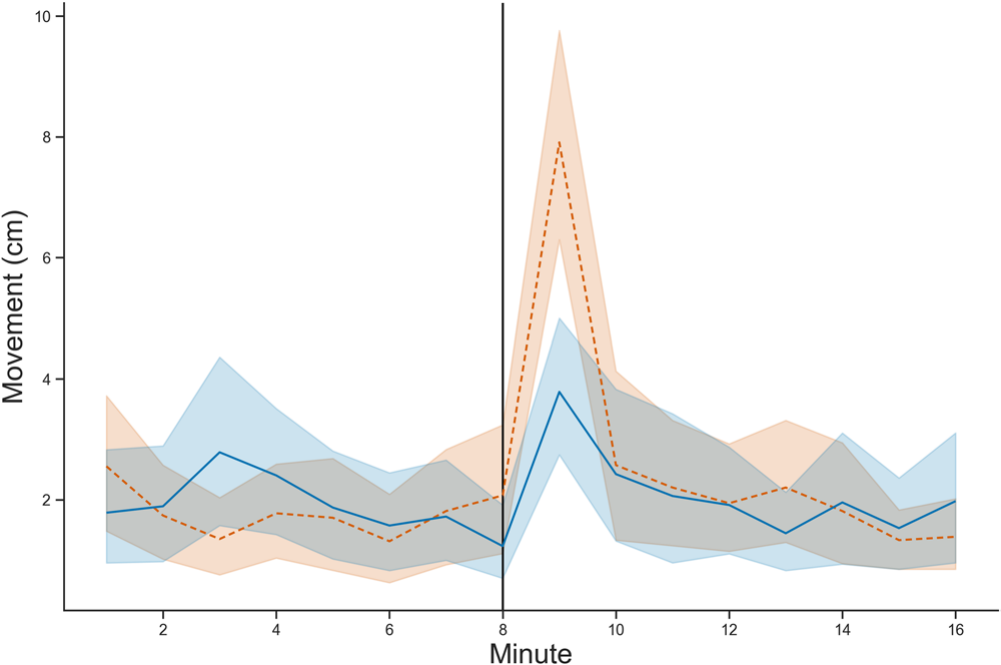
Movement of the shaken drones (orange dotted line, *n* = 54) and sham control drones (blue solid line, *n* = 47) during the 16 min observation. Intervention shown at minute 8 (vertical black line).

Shaken drones (*n* = 54) walked more than sham control drones (*n* = 47) after intervention with the magnetic shaker (lme, Shaken: 2.67 ± 0.34, Sham Control: 2.14 ± 0.39 cm min^−1^, Fig. 3). The type of treatment (shake or sham control), time since intervention, average speed of the bee prior to intervention, and interaction effect between treatment and time since intervention were all significant (lme, all *p* ≤ 0.001). In a post-hoc test, each minute was analyzed separately to look for a difference between the treatment groups. There was a significant difference in movement between the treatment groups in the first minute after intervention (Shaken: 7.91 ± 0.91, Sham Control: 3.79 ± 0.6 cm min^−1^, *p* < 0.001), but not during any of the other time periods.

Shaken drones were more likely to be fed, were fed more times, and were fed for longer than sham control drones in the 8 min after intervention with the magnetic shaker. Of the drones we observed, 55.6 ± 7% of the shaken drones were fed while only 34.0 ± 7% of the sham control drones were fed after intervention (glmer, *p* = 0.01). Shaken drones were fed 1.3 ± 0.2 times, while sham control drones were only fed 0.64 ± 0.23 times (*p* = 0.001). The number of times a drone was fed prior to intervention was a positive predictor of how many times he was fed after intervention (*p* < 0.001). Shaken drones were fed for 15.7 ± 4.2 s while sham control drones were fed for 4.2 ± 2.5 s (lmer, *p* < 0.001). The amount of time a drone spent being fed prior to intervention was a predictor of the amount of time he spent being fed after intervention (*p* = 0.014).

Shaken drones were more likely to be groomed and were groomed more often than sham control drones, but were not groomed for longer in the 8 min after intervention with the magnetic shaker. Of the drones we observed, 55.6 ± 7% of the shaken drones were groomed while 44.7 ± 7% of the sham control drones were groomed (glmer, *p* = 0.023). The number of times the individual was groomed prior to intervention was a positive predictor of whether or not he would be groomed after intervention (*p* < 0.001). Shaken drones were groomed 1.5 ± 0.2 times while sham control drones were groomed 1.0 ± 0.2 times (lmer, *p* = 0.002). The number of times a drone was groomed prior to intervention was a positive predictor of how many times he would be groomed after intervention (*p* < 0.001). Shaken drones were groomed for 22.1 ± 6.1 s while sham control bees were groomed for 11.4 ± 3.5 s after intervention, but this difference was not significant (lmer, *p* = 0.13). The amount of time a drone was groomed prior to intervention was not a significant predictor of how much time he was groomed after intervention (*p* = 0.18).

## Discussion

Vibrating a bee with the magnetic shaker elicited behavioral responses similar to those elicited by natural shaking signals in both workers and drones^6,12^. The shaker increased movement in workers and drones, increased the likelihood that a drone would be fed and groomed, increased the number of times drones were fed and groomed, and increased the duration of drone feeding by workers. The shaker did not significantly change the movement bearing of worker bees with respect to the exit, or increase the duration of drone grooming by workers. This shows that a 1.5 s vibration at a frequency of 15 Hz applied directly to a bee elicits behavioral responses from the recipient, regardless of other potential cues that could be present during a natural shaking event.

Artificially shaken workers walked significantly more than sham control workers after intervention with the shaker, and this effect lasted for the full 8 min observation period after intervention with the shaker. While artificially shaken drones walked significantly more than sham control drones, the increase in movement was immediate and brief, lasting less than 1 min. Boucher and Schneider (2009) found that shaken drones walked more than non-shaken controls immediately after being shaken. They did not, however, study how drone movement changed over time. We found that drones increase their speed after receiving a shaking signal, but return to their prior speed quickly if they are not shaken again.

Why do drones show a brief change in activity levels, whereas workers exhibit a long-lasting change? Workers are responsible for the vast majority of colony tasks, and the urgency of these tasks may change as colony and environmental conditions change^22^. A sustained increase in activity level may give workers time to reassess colony needs, and then act accordingly. Unlike workers, drones are not known to perform work within the colony. When drones are shaken, both naturally and artificially, they respond by seeking out workers to feed and groom them. This, presumably, does not require a broader assessment of colony and environmental conditions, but instead depends only on the drone finding a suitable worker. If feeding or grooming does not occur within the short time-window, then the drone can return to its original state.

Schneider *et al*. (1986) showed that shaken foraging-aged workers were more likely than non-shaken controls to move to the dance floor, where they encounter other foraging-related signals^11^. Nieh (1998) showed workers that received shaking signals were likely to either continue heading towards the hive exit, or to reverse their trajectory away from the exit^6^. We failed to detect any significant changes in the trajectory of workers after we intervened with the magnetic shaker. We also looked at the angle by which each worker changed her trajectory and found no significant difference between the shaken and sham control workers. This discrepancy between our results and those in the literature may be due to the differences in the ages of bees studied. In this study, we focused on middle-aged bees (15–17 days old), whereas Schneider *et al*. (1986) studied foraging-aged bees and Nieh (1998) randomly selected bees from the full age range of the colony. Middle-aged bees have a diverse behavioral repertoire within the colony and perform tasks throughout the nest including: comb building, offloading foragers, processing nectar, and guarding the entrance^22^. Bees of all ages are shaken^23^, and response thresholds to signals can vary among workers of different ages^2^. Given that middle-aged bees perform diverse tasks, they may not move in a clear pattern with respect to the exit, because the tasks they perform occur throughout the nest. These results present an intriguing area for future research: the response of an individual after receiving a shaking signal (whether they respond, how they respond, and the magnitude of their response) may depend on the age of the receiver, and their current task.

The shaking signal is used in conjunction with changes in foraging availability, as well as when the colony is swarming^6,14,15,24^. Therefore, the shaking signal plays a part in organizing colony-scale processes, but we know little about how the shaking signal may influence colony-level responses to changing conditions. It is known, however, that worker bees have a pronounced division of labor associated with worker age (temporal polyethism), and that shaking signals influence Juvenile hormone (JH), a key hormonal regulator of worker tasks^10,25^. Young workers start by performing relatively safe tasks within the nest, and transition to progressively riskier tasks as they age^1^. JH influences how quickly workers progress along the age-based behavioral sequence^25^. JH titers of shaken workers are increased compared to non-shaken controls after 15–30 min^10^, therefore, the shaking signal may play an important role in regulating division of labor within a colony. The shaking signal could provide a mechanism for older workers to influence the progression of younger workers along the behavioral sequence in response to changing colony needs.

Signals are often complex, and may have many pieces that converge to produce a behavioral reaction. The shaking signal is no exception. In a natural shaking event, the shaker grabs onto the recipient with her appendages and shakes vigorously. Shaking signals are frequently performed by foragers, so the shaker could also carry the scent of nectar and pollen from her recent foraging trips^6^. Furthermore, bees have a cuticular hydrocarbon profile that could act as a cue to the receiver of what type of worker is delivering the signal^26^. By mimicking shaking with a magnetic tool we were able to see if a vibration with the same frequency and duration, applied directly to the thorax of a recipient, elicited the same reaction as a natural shaking signal in the absence of other stimuli that would be present in a natural shaking signal. Our magnetic shaker eliminated these factors and still had significant effects on the behavior of both workers and drones, showing that the vibration of the honey bee shaking signal is responsible for much of its observed behavioral response.

Experiments studying the response of individuals to natural shaking signals are difficult and time-intensive. Previous studies have followed one individual at a time, waiting for the focal bee to be shaken by another bee. Under these natural conditions, it is easy to lose track of the focal bee if the view gets obstructed or the bee exits the hive. The ability to apply vibrations to mimic shaking signals will facilitate testing the function of this behavior in different contexts, castes, and across a range of ages. This tool will allow us to further investigate the role of the shaking signal in communicating information and coordinating labor among honey bee workers of different behavioral castes, and to investigate how this general signal functions to produce an appropriate colony-level response to changing environmental conditions.

## Methods

### Magnetic shaker design

**Figure 4:**
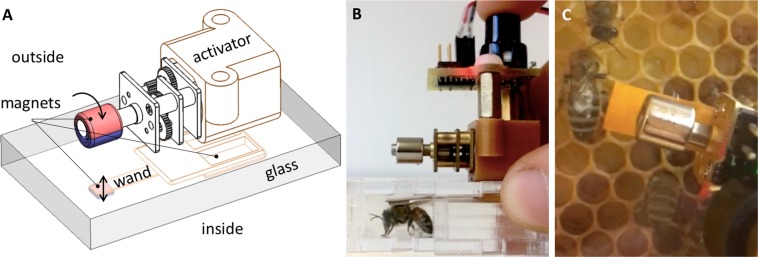
Magnetic shaker, composed of an activator on the outside of the hive and a vibrating wand on the inside to stimulate bees. (**A**) Design of the shaker. (**B**) Photo showing side view of the shaker when set up in a plexiglass arena with a bee. (**C**) Photo showing the magnetic shaker set up inside an observation hive.

To mimic the honey bee shaking signal, we designed a two part tool made of an activator and a wand (Fig. 4). The activator was placed against the glass on the outside of the observation hive, with the wand inside. Both pieces had two magnets that attracted them to each other through the glass, so that when we moved the activator, the wand moved with it. The activator consisted of a printed circuit board (Supplementary File 1) with a large push button trigger, a battery, and a brushed DC motor (6V Pololu geared 298:1) with a cylindrical, diametrically polarized, magnet glued onto the end of the motor shaft (K&J magnetics, part R424DIA). These parts were attached to a 3D-printed frame (Supplementary File 2). Beneath the motor were the two magnets that were placed against the glass to attract similar magnets in the wand. The wand consisted of a 3D-printed base (Supplementary File 3) with two attachment magnets glued into it, and a flexible metal arm with a 4.8 x 4.8 mm square magnet on the end. This magnet (K&J magnetics, part B3301) was the piece that came into contact with bees. It aligned with the magnet at the end of the activator shaft, but did not sit flush with the glass.

When the activator button was pressed, the motor spun at 15 Hz for 1.5 s. This spun the magnet on the end of the activator shaft which, in turn, vibrated the magnet on the flexible end of the wand back and forth, inducing mechanical shaking of the bee at 15 Hz for 1.5 s. This vibration is within the average duration and frequency range of naturally observed shaking signals^15^.

The wand was designed to approximate the shaking force exerted by one bee on another. The force exerted on a bee by the magnetic shaker is determined largely by the dipole interaction between the magnets on the activator shaft and the wand. For a given separation between the magnets, the force is maximized when the two magnets are oriented to repel. To estimate this maximum force at a typical separation of 10 mm, we oriented the activator magnet to repel the wand magnet and lowered the wand onto a scale. The maximum output force of the magnetic shaker measured 7.8 mN, which represents an upper limit. We estimated that the maximum force of a natural shaking signal, *F*_*bee*_ is on the same order of magnitude of a foraging-aged bee applying its entire body weight ( ~ 100 mg^27^) onto another bee, i.e., *F*_*bee*_ =  0.98 mN. The peak output force of the magnetic shaker is within an order of the magnitude of the calculated force from a natural shaking signal, from which we inferred that the magnetic shaker exerts forces comparable to natural shaking.

### Workers

We set up two honey bee observation hives with marked workers to study bee behavior before and after intervention with the magnetic shaker. We predicted that worker bees would walk faster and change their trajectories in response to the stimulus as they do in response to natural shaking signals^6^. We examined worker-shaker interactions in two queen-right, two-frame observation hives with approximately 2,500 bees at Cornell’s Dyce Lab for Honey Bee Studies, in Ithaca, NY during July and August 2018. In order to control for age in our study, we introduced cohorts of marked bees by incubating frames of capped, emerging brood from source colonies overnight (35 °C, 50% RH). We marked the bees that emerged overnight with a cohort-specific paint dot, and introduced 300 newborn workers to each of the hives. After introducing a cohort, we waited to observe them until they were 15–17 days old, in the middle-aged caste^28^. We chose this age because bees in the second and third week are the most common recipients of natural shaking signals, and bees in this age range are typically performing in-hive activities, so we were likely to see them in the colony during the day^29^.

The observation hives were setup with a single entrance/exit, with all bee traffic routed to a single side of the observation hive^6,15^). We only collected data on the exit side of the observation hives, where shaking signal activity primarily takes place^6^. The observer randomly chose whether to start with a shake (vibration) or sham control, and then alternated. To decrease the likelihood of stimulating a single bee multiple times, we collected data on a maximum of 10% of each cohort and chose our subjects randomly.

The observed side of the hive was set up with two grids, a 1 × 1 cm grid for tracking bee movement, and a grid that divided the hive into 9 squares for randomly selecting bees^6^. One of the 9 squares was chosen with a random coordinate generator, and the bee closest to the top left of the coordinate was followed. If there was no marked bee in the square, a new coordinate was generated. The initial position of the bee was marked on a transparency sheet covering the hive glass. We followed each bee for a total of 16 min (8 min before intervention, 8 min after). To measure the movement of the observed bee, we recorded each time the focal bee crossed a 1 cm gridline using a tally counter. At minute 8, we slowly moved the shaker to position it over the focal bee. Then, the shaker was either activated so that it vibrated for 1.5 s (shaken, *n* = 38), or was not activated (sham control, *n* = 39). This interaction was watched closely to make sure that the wand contacted the bee while vibrating. The treatment was not blind to the observer because the wand was activated with the press of a button, and could be heard and seen vibrating. The location of the bee during the intervention was recorded on the transparency.

We continued to follow the bee for 8 min after intervention. At the end of the observation period, the final position of the bee was marked. This gave us 3 positions for trajectory analysis of the bee: the location where we started observing, the location where we placed the magnetic shaker over the bee (shake or sham control), and the location where we stopped tracking the bee. In some trials we did not get all 3 of these locations successfully, so our sample size was lower than for the movement analysis (shaken: *n* = 32, sham control: *n* = 36). If the bee was lost after intervention, the time and final movement tally count were recorded and the last place the bee was observed was marked on the transparent sheet. If the bee was shaken by another bee while being observed, the time was noted and we stopped collecting data.

### Drones

We also studied drone behavior before and after intervention with the magnetic shaker, in the same observation hives. We predicted that drones would walk faster, get groomed and fed more times, and spend more time being groomed and fed after being shaken, as they do in response to natural shaking signals^12^. We incubated frames of drone comb from source field colonies overnight (35 °C, 50% RH). Within 24 hours, emerged drones were collected. We glued a numbered plastic tag to each drone so that we could sample each individual and prevent collecting data on a single drone multiple times. We introduced drones on 4 different days to establish a population of 60–100 drones in each colony. Workers have been shown to shake immature drones more than 2 times as often as mature drones^12^, so we timed our observations of worker-drone interactions when the drones were reproductively immature (2–10 days old^30^). We chose drones by randomly generating a coordinate indicating one of the 9 squares that divided the hive, and choosing a drone within that square. If there were no drones that had not been previously observed, a new coordinate was generated until a qualified drone was found. During the period of observation (16 min: 8 before intervention and 8 after), we kept track of the distance the drone walked each minute, and the number of times a drone was fed or groomed and for how long, using a stopwatch and tally counter. We intervened at minute 8 by placing the magnetic shaker so that it was contacting the focal drone and either activating it (shaken, *n* = 54) or not (sham control, *n* = 47).

### Statistical analysis

All statistical analyses were performed in R version 3.5.1^31^. To compare the movement of workers and drones that were shaken compared to those that were not, we built linear mixed-effects models^32^. We used an autoregressive lag-1 correlation structure in order to reduce the confounding effects of temporal autocorrelation. We built these models to test for differences in movement of bees that were shaken by the wand and those exposed to the sham control, with colony as a random effect, and time since intervention, average movement of the bee in the 8 minutes prior to intervention, and the time of day (morning or afternoon) as fixed effects. We square-root transformed the response variable (movement, in cm).

The trajectories of the workers with respect to the hive exit before and after intervention were analyzed separately for shaken and sham control workers using Moore’s test for paired circular data^33^. The angle by which each bee changed its trajectory was analyzed using an unpaired Watson two test from the circular package in R^34^.

For the drone behaviors, we recorded the number of times the drones were fed or groomed by workers, and how long each of these interactions lasted. We then created linear mixed-effects models for: the number of times drones were fed, the number of times drones were groomed, time spent being fed, and time spent being groomed. Each of these models was built using treatment (shake or sham control), the number of times (or for how many sec) the bee was fed or groomed prior to the intervention, the age of the drone, and the date as fixed effects. The colony was treated as a random effect. To make models of the number of times the drone was fed, the time spent being groomed, and the time spent being fed, we square-root transformed the response variable. To analyze the likelihood that a drone would get fed or groomed after our intervention, we used generalized linear mixed models with a binomial distribution. In these models we used treatment group, the number of times the bee was fed or groomed prior to our intervention, and the age of the drone as fixed effects, and colony as a random effect.

## Supplementary information

Supplementary Information 1.

Supplementary Information 2.

Supplementary Information 3.
